# Phenology of Scramble Polygyny in a Wild Population of Chrysolemid Beetles: The Opportunity for and the Strength of Sexual Selection

**DOI:** 10.1371/journal.pone.0038315

**Published:** 2012-06-25

**Authors:** Martha Lucía Baena, Rogelio Macías-Ordóñez

**Affiliations:** Red de Biología Evolutiva, Instituto de Ecología A.C., Xalapa, Veracruz, Mexico; University of Plymouth, United Kingdom

## Abstract

Recent debate has highlighted the importance of estimating both the *strength of sexual selection* on phenotypic traits, and the *opportunity for sexual selection*. We describe seasonal fluctuations in mating dynamics of *Leptinotarsa undecimlineata* (Coleoptera: Chrysomelidae). We compared several estimates of the opportunity for, and the strength of, sexual selection and male precopulatory competition over the reproductive season. First, using a null model, we suggest that the ratio between observed values of the opportunity for sexual selections and their expected value under random mating results in unbiased estimates of the actual nonrandom mating behavior of the population. Second, we found that estimates for the whole reproductive season often misrepresent the actual value at any given time period. Third, mating differentials on male size and mobility, frequency of male fighting and three estimates of the opportunity for sexual selection provide contrasting but complementary information. More intense sexual selection associated to male mobility, but not to male size, was observed in periods with high opportunity for sexual selection and high frequency of male fights. Fourth, based on parameters of spatial and temporal aggregation of female receptivity, we describe the mating system of *L. undecimlineata* as a scramble mating polygyny in which the opportunity for sexual selection varies widely throughout the season, but the strength of sexual selection on male size remains fairly weak, while male mobility inversely covaries with mating success. We suggest that different estimates for the opportunity for, and intensity of, sexual selection should be applied in order to discriminate how different behavioral and demographic factors shape the reproductive dynamic of populations.

## Introduction

What should we expect from an estimate of the strength or intensity of sexual selection? The answer clearly depends on the assumptions behind each estimate and on the data we use, which in turn depend on the ecological context of the population. The strength of sexual selection acting on one or several phenotypical traits may be estimated using selection differentials (*β*) (reviewed by Jones [1]). In order to do this, data on reproductive success and the phenotypic traits likely under selection should be available. However, a candidate trait is not always easy to identify when a mating system is studied for the first time. Furthermore, data on reproductive success is not always available.

The strength of sexual selections is also expected to be strongly influenced by ecological factors, and several estimates have been proposed considering such factors (reviewed by Jones [1] and Klug *et al.* [2]). Specifically, the resources that promote aggregation of potential mates in space or time should have a strong effect on the reproductive strategies of a population. Ecological factors such as food availability or oviposition sites, among others, may influence female aggregation patterns and therefore, distribution of reproductive success among males [3], [4] which will all impact the upper limit for the strength of sexual selection, *i.e*., the opportunity for sexual selection (*I_s_ or I_mates_*), which in itself is a useful descriptor of the mating system and the potential for sexual selection to act on one or many phenotypic traits (for a recent debate on this see [2], [5], [6]). Both the strength of, and the opportunity for, sexual selection may provide different and potentially complementary information on the reproductive ecology of the population, but they have rarely been compared or contrasted on the same wild population.

All parameters of sexual selection also depend on the time scale at which the data are analyzed, the sampling design and the assumptions behind the analysis [7]. In short time scales (*e.g*., daily estimates) we obtain a snapshot of only a few reproductive events, if any, per male, and the maximum resulting degree of female (selectivity) aggregation around males is constrained, and therefore the variance in male mating success is frequently low. Thus the influence of operational sex ratio may be strong. On the other hand, if data are accumulated over long periods (*e.g*., the whole reproductive season) and then analyzed together to produce a single value, we obtain overall values but lack information on the temporal variation of such parameters and potential relationships with ecological factors. Furthermore, when data from the whole season are used to obtain a single estimate, all males are assumed to be present throughout the whole period which frequently is not the case. Thus, the effect of unsuccessful males in the analyses may be overestimated.

Recent research [8], [9] suggests that average estimates of selection over long periods may mask relevant fluctuations in the intensity of selection on different phenotypes. However, such studies have been carried out on species with long lifespan, usually with one relatively synchronous reproductive event per mating season. Polygynous species with short life spans relative to their reproductive season may be more suitable to explore how demographic factors such as fluctuations in operational sex ratio or density affect reproductive opportunities and, therefore, sexual selection at smaller scales [10], [11]. In order to do this, we suggest adopting an intermediate time scale that reflects relevant fluctuations of demographic parameters and female receptivity within the mating season, but also relevant variance in male mating success accumulated over time.

Time should be especially important for species in which the prospect of breeding declines within the season. If breeding prospects diminish because of a change in operational sex ratio or mate availability, choosiness should also decrease with time [12]. However, operational sex ratio reveals only the possible competitive circumstances at a particular time and place, and this does not allow specific predictions on how sexual selection operates [2], [4], [13], [14]. When female receptivity is clumped in time or space, a better estimate may be the between-time-interval covariance in individual male mating success, which shows how consistent certain males are accumulating mating events over time. If this covariance is low (i.e. the mating success of a given male in one day does not predict the mating success of the next day), then there will be little selection pressure to influence female choosiness (see *Cov*_(phen)_ below [4]).

The opportunity for selection, calculated as the variance in absolute total fitness divided by the square of mean total fitness, was suggested by Crow [15] as a method to estimate the intensity of (natural) selection and modified by Wade [16] to estimate the intensity (strength) of sexual selection as *I*_mates_ or *I*_s_ depending on whether mating success or reproductive success is used (reviewed by Jones [1]). *I*_mates_ is the ratio of the variance to the square mean number of mating events and reflects the maximum potential strength of sexual selection in a particular population [16]–[19]. Subsequently, Wade [20] suggested that an index of female spatial aggregation (*female spatial mean crowding* or m*) is equivalent to *I*_mates_ under resource defense polygyny when sex ratio equals 1. More recently, Shuster and Wade [4] suggested three ways to estimate the opportunity of sexual selection (*I*_mates_) based on the qualitative model proposed by Emlen and Oring [3], including the effect of unequal sex ratios and the concept of female spatial and temporal aggregation.

The first may be used when only the distribution of copulas in time and space (or around males) is available and we will refer to it as *I*_mates_ from now on. It is based on the average number of receptive females per patch (resource defense polygyny) or per successful (mated) male (*m*) (any form of polygyny), as well the variance in mating success ([4], equation 2.13). The second alternative, *I*_mates(adj)_ ([4], equations 2.15 and 2.28), incorporates the concept of female spatial aggregation (m*) and sex ratio (*R*) in the estimate of the opportunity for sexual selection. The third way to estimate the opportunity for sexual selection suggested by these authors, which we will refer to as *I*_mates(phen)_, may be used when detailed information on receptive phenology of each female is available. In other words, it requires detailed information on the individual identity of copulating males and females [14], thus we can analyze variation in female reproductive synchrony as female receptivity changes in time. Therefore, *I*_mates(phen)_ estimates the relative ability of individual males to obtain mates in time based on sex ratio (*R* [4], equations 3.16 and 3.18). The covariance among time intervals in male mating success (*Cov*_(phen)_) may also be estimated when individual identity is available for males, indicating how consistently successful or unsuccessful individual males are from one day to the next (see Materials and Methods and File S1 in Supporting Information). When this covariance is high, reproductive competition within intervals is amplified over the entire season, thus increasing the value of *I*_mates(phen)_. This approach based on sex ratio is better than instantaneous estimates of operational sex ratio (R_o_ in [3]) that do not consider such covariance, often leading to overestimates of the opportunity of sexual selection [4].

Other indices of female monopolization include the index of resource (females) monopolization (*Q*), Morisita’s index (*Iδ*) and the standardized Morisita index (*Ip*) [1], [21], [22]. These, however, consider the spatial distribution of females among males, and attempt to consider the random expectations on mating success, but not the temporal distribution of females. *I*_mates_ [4] involves not only the spatial distribution of females (m*) but their temporal distribution as well (t*): the reproductive phenology of receptive females among males. Furthermore, unlike the cited indices, *I_mates_* is the only measure that has a formal tie to mathematical sexual selection theory [23] and effectively integrates the contributions of mate choice, social interactions, mate monopolization and other factors affecting mating patterns in a single value, thereby providing a concise description of the distribution of fertilizations, and it is this variance in mating success that drives sexual selection [1], [4], [24]–[26].

When data is available to estimate female aggregation in time (*mean temporal female crowding* or t*) and in space or around males (*mean spatial female crowding*, m*), we have the opportunity to visualize the reproductive dynamic of a mating system since slight changes in spatial distribution over time may result in rapid changes in the value of the opportunity of sexual selection ([4] chapter 3, [14]). Thus, when females are aggregated in space either around specific sites or around males, the highest limit in the value of *I*_mates_ is directly proportional to the variance in reproductive success. The more aggregated the females are in space or around males (higher m*), the higher the opportunity for sexual selection since one or few males may defend and mate with all females in the population. On the other hand, when females are aggregated in time (higher t*), the opportunity for sexual selection is low since the ability of one or a few males to mate multiply is low. The combined effect of m* and t* on the opportunity for sexual selection can be represented as a three dimensional space in which Emlen and Oring’s [3] descriptive model of mating systems may be quantified [4]. Thus, mating systems that do not clearly fit any of the fixed categories suggested by these authors may be quantitatively defined and compared, or the mating system of the same population may be compared at different points in time.

According to Klug *et al*. [2], the opportunity for sexual selection is a poor predictor of the intensity of sexual selection especially when mate monopolization is strong, since selection is not quantified in relation to phenotypic traits (although see [5]). Furthermore, these estimates depend on (and thus are said to be “biased” by) mean male mating success and number of males [21], [23]. Here, we contrast observed values of different *I*_mates_ estimates against null models (under random mating); a desirable practice [2], [23] rarely applied in this context (6, see [27] for an exception). We will suggest that the relationship between the observed and the randomly expected value of the opportunity for sexual selection provides an assessment of the effect of the variance in male mating success on the opportunity for sexual selection independently of male average mating success and number of males (density). Furthermore, this allows “fair” comparisons between populations or between time periods even when they differ in number of males, average male mating success or sex ratio.

In the context of female spatial and temporal distribution, when females are spatially dispersed and breed synchronously, most males are expected to be able to mate [3], [4]. Under these circumstances, even if there is female preference for any phenotypic trait (*e.g*., body size has been shown to influence mating success in insects [28]–[32]) the value of *I*_mates_ is unlikely to increase due to weak sexual selection and thus such trait may not provide a mating advantage. A first aim of the study was to test this prediction under field conditions by using a mating differential recently suggested by Jones [1] on one morphological (size) and on one behavioral (mobility) male trait in a field population of *Leptinotarsa undecimlineata* (Stål).

When female receptivity is less synchronic and more aggregated around some males we expect more fights among males. Thus, our second aim was to describe changes in frequency of male fights throughout the season and find potential relationships with the reproductive phenology of the population.

Our third aim was to explore the opportunity for sexual selection (spatial and temporal distribution of female receptivity around males, sex ratio and variance in male mating success) put forward by Shuster and Wade [4] in *L*. *undecimlineata*. Although Emlen and Oring [3] suggested the use of temporal and spatial aggregation parameters to define mating systems, only recently Shuster and Wade [4] suggested a method for doing so quantitatively, and few, if any attempts have been carried out to apply such method on field populations. Furthermore, we know of only one previous study which explored the relationship between *I*_mates_ and spatial distribution of females [33]. A fourth aim was to compare the observed fluctuation in the strength of and opportunity for sexual selection with values assessed for the whole season in order to assess the shortcomings of ignoring such temporal fluctuation.

As in many other chrysomelids [34], [35], previous studies and preliminary observations suggest a *scramble competition polygyny* [29] in the studied population of *L. undecimlineata*. Males are aggressive sometimes to other males in copula, but no female or any other reproductive resource is monopolized (*e.g*., [36], [37]). Although scramble competition polygyny is probably the most common mating system among insects [29], [34], it has received much less attention [38] and may be harder to characterize when compared to more commonly studied mating systems such as resource defense or lek polygyny. Thus, we quantitatively defined the mating system of *L. undecimlineata*, and analyzed the relationship between male size and mobility, fighting behavior, mating success, and different estimates of the opportunity for and intensity of sexual selection.

In short, we aimed to describe the mating system of *L. undecimlineata* and assess the circumstances under which estimates of the *strength* of sexual selection would correspond to estimates of the *opportunity* for sexual selection, or to actual peaks of male-male competition for mates, including the use of null models and comparing a whole-season to a phenological approach to data analyses.

## Materials and Methods

### Study Site

The study was carried out in a secondary forest that supplanted a pasture that had been abandoned for seven years. The site is situated next to a fragment of cloud forest at “El Riscal”, in Central Veracruz, Mexico (19°28′56′′N, 96°59′48′′W, 1595 asl) between July 21 and November 7, 2004. Mean annual temperature is 20°C (Min  = 12°C, Max  = 34°C) and precipitation fluctuates between 2000 and 3000 mm [39], [40]. The population of *L. undecimlineata* was found on a patch of 75 adult plants and 278 nonsexual juvenile plants of *Solanum lanceolatum* Cav. and eight adult plants of *S. chrysotrichum* Schltdl, covering approximately 400 m^2^. No special permits were required as no samples were collected and the field site belongs to one of the authors (RM).

### Study Species

After spending the period between mating seasons underground, adults emerge in the summer, although a few may spend the whole year on the host plant [36]. They use *S. lanceolatum* and *S. chrysotrichum* as larval and adult feeding resource, as oviposition resource for females, and as mating site [36], [41]. They are only found on these two plants and not on any other herbaceous or arboreal species. However, all adult host plants seem equally suitable for males and females, and all leaves within each plant seem to be used as either mating, feeding or oviposition site. During the second half of the season, larvae have consumed most of the foliage of adult host plants, and adults move to much shorter non reproductive young plants (Baena and Macías-Ordóñez, unpublished).

**Figure 1:**
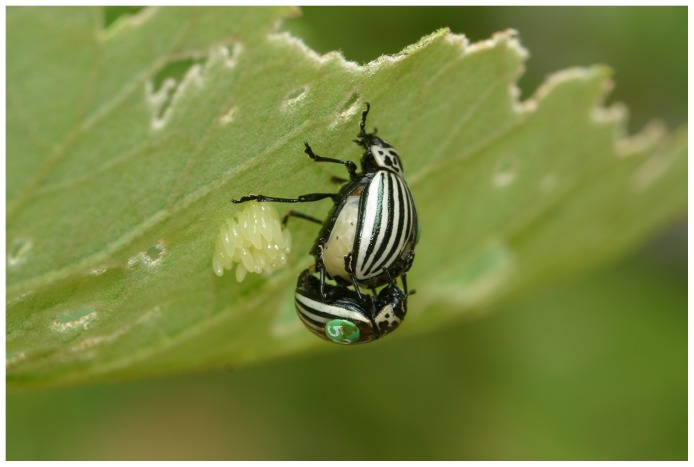
Mating and oviposition in *L. undecimlineata*. Male and gravid female in copula (notice aedeagus intromission) next to a batch of eggs recently laid by the female under a leaf of the host plant *Solanum lanceolatum*.

The following facts, based on our observations, provide some general natural history of this species, given the scarcity of field studies [36]. Many individuals are present for only some periods of the reproductive season. Both males and females mate with different mates, and females may mate repeatedly with same male. Females frequently oviposit after mating and males usually stay on or near the female, seemingly guarding and/or courting her, and then copulate again. This mating-oviposition sequence with the same male may be repeated up to 13 times during up to 4 hours (Figure 1). However, most of the time (around 80%) females mate only once with one male. Males seem to court female before and after mating and we did not observe any male - female interaction that suggested forced copulation. Although females are clearly larger than males, no other secondary sexual character is evident in either sex.

### Behavioral Records and Sampling Design

Host plants were individually marked with aluminum tags. Beetles were individually marked using standard plastic queen honey bee tags (2 mm in diameter, Figure 1). The right or left of the elytron, depending on sex, was lightly scratched with sand paper and then the tag was glued with Instant Krazy Glue®, and returned to the same leaf or stem in which they were collected. All efforts were made to minimize handling time. After initial measuring and marking, individuals were never handled again. A total of 660 males and 377 females were tagged. Each individual was measured using a caliper (±0.01 mm). We recorded body length excluding the head (the head was not included to reduce measuring error since individuals moved and even retracted the head when handled), maximum abdomen width and abdomen height at the first abdominal segment. Body size was defined as body volume, estimated using an ellipsoid approximation [42].

A *mount* was recorded when a male climbed on a female, standing parallel to the female’s dorsum, and remaining still for at least one minute without aedeagus intromission. A *copula* was recorded if a mount was observed and aedeagus intromission (Figure 1) was recorded to last at least 5 minutes. A male was considered *successful* if at least one copula was recorded during the time period analyzed (as explained below, the reproductive season was divided in four periods). Males observed only eating, walking, mounting or standing still during the same period were considered unsuccessful. Females were considered receptive in a given time period if they copulated at least once. According to Bonduriansky [43], a female is mature in reproductive terms if she copulates. A *fight* was recorded when a male climbed on another male, parallel to the other male’s dorsum and seemingly attempted to bite the confronted male while the later seemingly attempted to dislodge the attacker using his legs. In most cases, fights occurred when a male climbed on a mounting or mating couple, frequently interrupting the sexual interaction. Males usually remained fighting on top of the female, eventually climbed down and oriented ventrally to each other with their legs intermingled, usually within two cm from the female. In this position, they frequently rolled down the host plant leaf or stem to the ground where they separated and climbed back on the same host plant. No fights were ever recorded in the absence of females.

All behavioral records were carried out in focal observations of 30 min on plants hosting at least one individual of each sex, between 8∶00 and 18∶00 Central Standard Time (GMT-6). Censuses were carried out by visual inspection of each plant along the same route, but alternating the starting and ending point every day. However, given the great variability of time allocated to each host plant depending on the number of individuals and their behavior on each, plants in the middle of the route varied greatly on the actual time of the day they were sampled, thus any effect of time of day is negligible. Given that the longest distance between any two host plants was less than 30 m, two persons could easily census and monitor all plants throughout the day, record the great majority of behavioral patterns, and carry out the 30 min focal observations on mating pairs.

**Figure 2:**
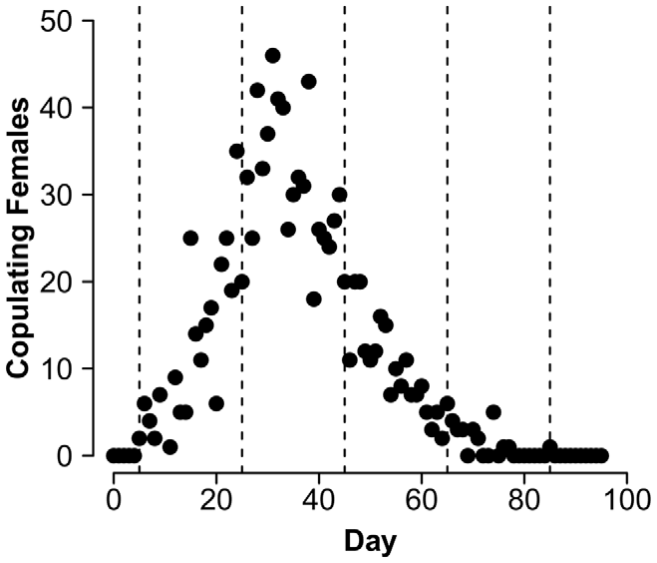
Number of daily observed copulating females in a population of *L. undecimlineata*. Observations were carried out between July 21 (day 0) and November 7 (day 95), 2004. Dashed lines define four 20-day periods between days 6 and 85 (in which the first and last mating couples were observed), used to analyze temporal fluctuation in the reproductive dynamics of the population.

Although individuals were observed on the host plants for 95 days, receptive females (in copula) were only observed from day 6 (July 26) to day 85 (October 23), thus an 80 day mating season was considered for analysis. The season was divided in four 20-day periods based on clear changes in the reproductive dynamics observed in descriptive data, such as a surge in reproductive activity between days 26 and 45 (Figure 2). This seemed to be an adequate temporal scale to analyze the effect of changes in female aggregation in this population since the variance of the number of receptive females per period was larger than the mean (**V_m_** = 3.28; **m** = 0.95), as suggested by Shuster and Wade [4]. All analyses were performed for each of those periods and for the whole 80 day season in order to assess temporal fluctuations in the reproductive dynamics of the mating system.

### Data Analysis

We obtained the standardized mating differentials *m*’ [1] for each period as comparable estimates of the effect of male size (volume) and mobility (number of plants occupied over total number of days observed) on mating success in order to assess the intensity of sexual selection related to a morphological and a behavioral trait. A bootstrap resampling procedure (10,000 resamplings) was carried out using the *boot* library of the R statistical package [44], in order to estimate 95% confidence intervals of the observed values of *m’*. The same procedure was applied to obtain 95% confidence intervals for different estimates of *I*_mates_ and their parameters described below. Although both *m’* and *I_mates_* do not require actual reproductive success data, they safely assume at least some correlation between mating and reproductive success, and their predictive power of the genetic consequences on the population depends on the strength of such correlation (i.e. the Bateman gradient) [1], [4], [45].

We estimated the first index of the opportunity for sexual selection (*I*_mates_) using the number of mating events per male. We calculated *I*_mates_ based on the average number of females per successful male (*m*), as well as two components of male mating success: the variance among successful mates, those that mate at least once (*V*_harem_), and the variance between successful and unsuccessful males (*V*_mates_). The opportunity for sexual selection among successful males only (*I*_harem_) was also estimated (see File S1 in Supporting Information). Furthermore, we performed the adjusted estimate of *I*_mates_ weighted by spatial mean crowding (m*) and sex ratio (*R*), *I*_mates(adj)_. Female spatial mean crowding around males (m*) is the ratio of average number of females to *V*_harem_. Sex ratio (*R* [4]) was estimated as number of copulas over the total number of males. Since we had detailed temporal information, and *I*_mates_ does not reflect temporal variation in male relative individual ability to obtain mates [4], we also estimated the total opportunity for sexual selection based on phenology of female receptivity, *I*_mates(phen)_, which reflects changes in female receptivity in time and their effect on male mating success. *I*_mates(phen)_ was obtained as the sum of *I*_sexratio_, the opportunity for selection caused by temporal variation in sex ratio and *I_mates(t)_, caused by the variance in mating success among males averaged over time and weighed by sex ratio, and then substracting *I_males(k)_, caused by the average temporal variance in mating success per male. Furthermore, we estimated the covariance among time intervals in male mating success (*Cov*_(phen)_). The detailed procedure to estimate each parameter may be found in chapters 2 and 3 of [4], and in File S1 of Supporting Information.

We developed null models for all parameters. We shuffled the matrix of mating events 5000 times, estimating all parameters in each case in order to assess the random value of each parameter (the average of all 5000 replicates), given the number of mating females and males in each period (and thus the operational sex ratio). Such procedure was applied to each time interval and to the whole reproductive season. The R [44] code to estimate observed parameter values and all null models is included in Files S2 of Supporting Information (and the required databases in Files S3, S4, S5, S6, S7).

Additionally, we were interested in the relationship between abundance of individuals (number of individuals per plant), occupied host plants and male aggression throughout the season in order to compare with results of the previous analyses. First, in order to assess if the number of individuals per plant depended on period or differed between sexes, we performed a Generalized Linear Model (GLM) using a proportional response variable with binomial error on abundance of individuals per plant as dependent variable, sex and period as factors, and occupied plant abundance as covariable. The response variable was a two vector object made of the number of plants with at least one male and one female per day, and the difference between total number of individuals observed each day and the number of plants on such days (see chapter 16 in [46]). Second, in order to assess whether the number of fighting males depended on male, female or plant abundance, we performed a GLM with Poisson error distribution, on number of fighting males as dependent variable, period as factor, and male, female and plant abundance as covariables. Third, we were interested in how the frequency of fights was related to the number of fighting males, male and female abundance. Thus we performed a similar analysis in which number of fights was the dependent variable and number of fighting males was an additional covariable. In all cases we obtained the minimal model by a process of model reduction. The R statistical package [44] was used for all analyses.

## Results

### Individual Abundance Throughout the Season

**Figure 3:**
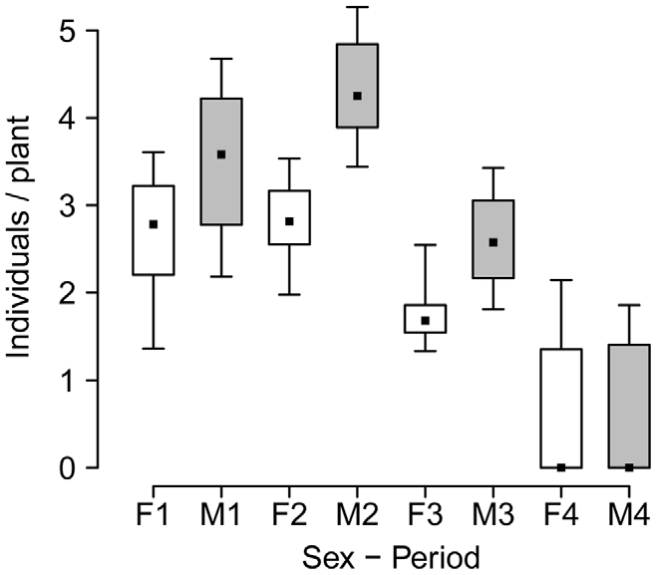
Male and female abundance over the mating season of *L. undecimlineata*. Number of males (shaded boxes) and females (open boxes) per plant observed over the four periods of the mating season (n = 20 days in all periods). Median, quartiles and extreme values are represented. Males are significantly more abundant than females overall, and this difference is significantly greater in period 2 (Table 1).

The minimum model for the analyses on abundance of males and females of *L. undecimlineata* per plant explained 79% of the deviance and identified *Sex*, *Period*, and the covariable (occupied *Plants*), as significant factors; *Period* by itself explained 45% of the model deviance. Additionally, *Sex*:*Period* and *Period*:*Plants* were significant interactions (Table 1). Abundance per plant peaked in period 2 and had its lowest value in period 4 while periods 1 and 3 had similar abundance. Overall, there were more males per plant than females, and the significant interaction between sex and period revealed that males were even more abundant than females in period 2 (Figure 3).

**Table 1:** Minimal GLM model for individual abundance per plant in a population of *L. undecimlineata.*

Factor	d. f	Deviance	Explained deviance	P
Sex	1	170.48	25%	<0.001
Period	3	303.99	45%	<0.001
Plants	1	19.04	3%	<0.001
Sex:Period	3	18.05	3%	<0.001
Period:Plants	3	20.72	3%	<0.001
*Null*	*137*	*671.01*		
*Residual*	*126*	*138.71*	*21%*	

See Figure 3.

Out of 660 males and 377 females recorded, 635 (96.2%) males and 343 (91%) females were recaptured at least once, and 404 (63.6%) males and 262 (76.4%) females copulated at least once in the mating season. Over one third of the males, 231 out of 635 (36%), did not mate at all; 178 (28%) mated once, while 226 (35.6%) copulated twice or more.

### Mating Differentials on Male Size and Mobility

**Figure 4:**
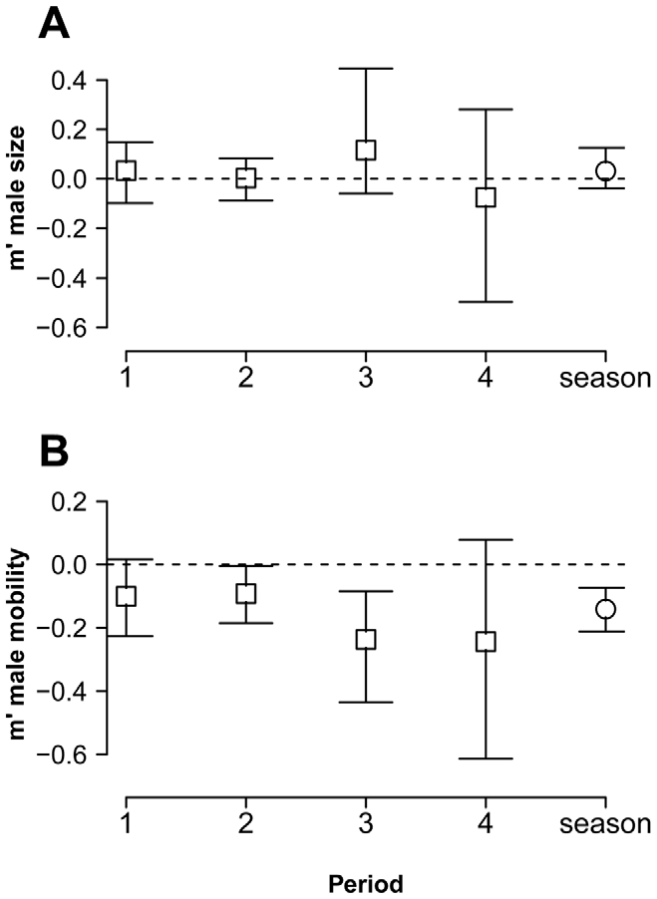
The strength of sexual selection in *L. undecimlineata*. Standardized mating differentials (*m*’) and bootstrap 95% confidence intervals for male size (**A**) and mobility (**B**) in each period and the whole reproductive season. None of the standardized mating differentials on male size differed from random expectations (dashed horizontal line at y = 0) in any period or the whole season. Male mobility (plants occupied per day observed) showed negative values significantly different from random expectations in periods 2 and 3, and the whole season. Males that moved less had higher mating success.

None of the standardized mating differentials (*m*’, [1]) on male size differed from random expectations (0 covariance between male volume and male mating success) in any period or the whole season (Figure 4A). However, male mobility (plants occupied per day observed) showed negative values significantly different from 0 for periods 2 and 3, and for the whole season. In such periods, males that moved less had higher mating success (Figure 4B).

### The Opportunity for Sexual Selection Throughout the Mating Season

**Figure 5:**
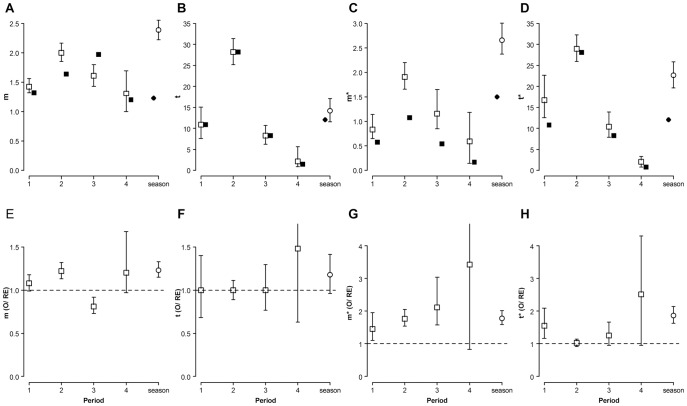
Spatial and temporal female crowding in *L. undecimlineata*. Estimates and bootstrap 95% confidence intervals of average mating success (mated females per successful male, *m*), number of mated females per time period (*t*), mean spatial female crowding around males (m*) and mean temporal female crowding (t*) in each period (squares) and for the whole season (circles). **A–D**) observed values (open markers) and values expected under random mating (solid markers). **E–H**) the ratio between observed and randomly expected values (O/RE) from the null model of each estimate. The dashed horizontal lines at y = 1 represent no difference between observed and randomly expected values (O/RE  = 1).

**Figure 6:**
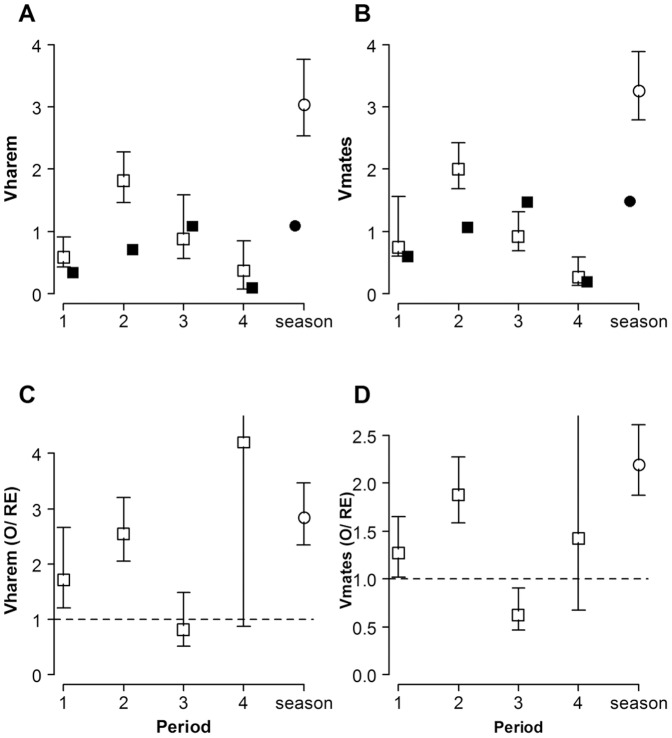
Variance in male mating success in *L. undecimlineata*. Estimates and bootstrap 95% confidence intervals of variance in mating success among successful males (*V*_harem_) and among all males (*V*_mates_) in each period (squares) and for the whole season (circles). **A–D**) observed values (open markers) and values expected under random mating (solid markers). **E–H**) the ratio between observed and randomly expected values (O/RE) from the null model of each estimate. The dashed horizontal lines at y = 1 represent no difference between observed and randomly expected values (O/RE  = 1). Variances in male mating success among successful males (*V*_harem_), and among all males (*V*_mates_) were significantly higher than expected under random mating for period 2, barely higher for period 1, and not different or even significantly lower than random expectations in period 3.

**Figure 7:**
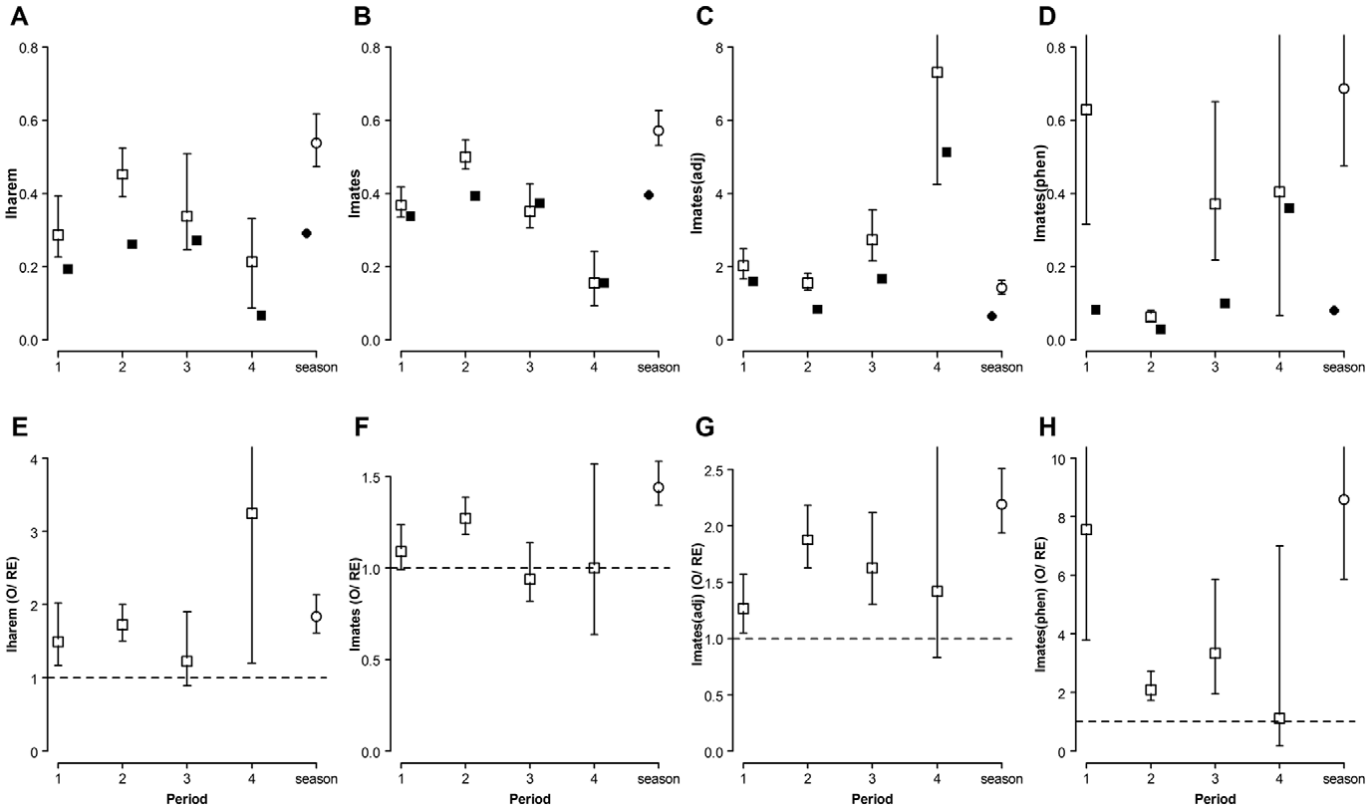
The opportunity for sexual selection in *L. undecimlineata*. Estimates and bootstrap 95% confidence intervals of the opportunity for sexual selection among successful males (*I*_harem_), the opportunity for sexual selection (*I*_mates_) derived from the relationship between the number of mated females per successful male (*m*), the variance in mating success among successful males (*V*_harem_) and among all males (*V*_mates_); its value adjusted (*I*_mates(adj)_) to sex ratio (R) mean spatial female crowding around males (m*), and the opportunity for sexual selection derived from female receptive phenology (*I*_mates(phen)_) in each period (squares) and for the whole season (circles). **A–D**) observed values (open markers) and values expected under random mating (solid markers). **E–H**) the ratio between observed and randomly expected values (O/RE) from the null model of each estimate. The dashed horizontal lines at y = 1 represent no difference between observed and randomly expected values (O/RE  = 1). All four estimates of *I* for period 2, and two (*I*_mates(adj)_ and *I*_mates(phen)_) for period 3, are significantly higher than random expectations. The opportunity for sexual selection for both estimates that factor in sex ratio (*I*_mates(adj)_ and *I*_mates(phen)_) resulted in significantly higher randomly expected values for periods 1, 2 and 3. Relatively fewer mating events in period 4 due to lower density in the population and a decrease in reproductive behavior resulted in much larger confidence intervals.

Figures 5, 6 and 7 show observed and randomly expected values (and bootstrap 95% confidence intervals) for ten of the previously described parameters associated with the opportunity for sexual selection in *L. undecimlineata*: average mating success of successful males (*m*), average number of mated females per time interval (*t*), mean spatial (m*) and temporal (t*) female crowding, variance in mating success among successful males (*V*_harem_), and variance in mating success among all males (*V*_mates_), the opportunity for sexual selection among successful males (*I*_harem_), the opportunity for sexual selection derived from the variance among males in mate number (*I*_mates_), its value adjusted to sex ratio and female spatial mean crowding around males (*I*_mates(adj)_), and the opportunity for sexual selection derived from female receptive phenology (*I*_mates(phen)_). For brevity we highlight only the most relevant patterns comparing among periods.

#### Periods 1 and 3

The observed values for all ten parameters were not significantly different between periods 1 (days 6 to 25) and 3 (days 46 to 65) according to their 95% bootstrap confidence intervals (open squares in Figures 5A–D; 6A, B; 7A–D). However, the expected values under random mating (solid squares in the same figures) for m, *V*_harem_, *V*_mates_ and *I*_harem_ follow a different tendency when compared to the observed values (Figures 5A, 6A,B and 7A). On the other hand, the randomly expected values in the remaining parameters (*t*, m*, t*, *I*_mates_, *I*_mates(adj)_ and *I*_mates(phen)_) follow the same tendency of the observed values, and in some cases completely match them (Figures 5B–D, and 7B–D).

In order to quantify the extent to which all these parameters differ from their expected value under random mating (and to what degree such difference significantly differs among periods), Figures 5E–H, 6C, D, 7E–H show the ratio between the observed and randomly expected values (O/RE) and their 95% confidence interval. The dashed line at *y* = 1 represents an observed value not significantly different from expectations under random mating. Some previously hidden differences emerge between periods 1 and 3. In the case of *V*_mates_, for instance, the observed values were not significantly different between periods 1 and 3, but the value in period 1 is significantly higher than random expectations, and significantly lower in period 3 (Figure 6B,D). Conversely, in the case of *I*_mates,_ once divided by their respective randomly expected values, periods 1 and 3 remain not significantly different among them, and their confidence intervals overlap with the broken line thus none of the observed values for these two periods is significantly different from expectations under random mating (Figure 7F).

#### Period 2

In the case of period 2 (days 26–45), most of the observed values differed significantly from period 1, 3, or both. It is worth noting that the observed values for period 2 are higher than in periods 1 and 3 in all but two estimates, *I*_mates(adj)_ and *I*_mates(phen)_, which show the opposite pattern (Figure 7C,D ). However, when divided by their expected values under random mating, a different story emerges for most parameters. In the case of *t*, for instance, the O/RE ratio of period 2 is not significantly different from periods 1 and 3 nor from random expectations (Figure 5F). In the case of m* or *I*_harem_ there is no significant difference between O/RE ratios of periods 1, 2 and 3, but with one exception (*I*_harem_ for period 3), they are significantly higher than expectations under random mating (Figures 5G and 7E). In most remaining estimates, however, O/RE ratios for period 2 are significantly higher than random expectations, and significantly different from either period 1 or 3, although never from both.

#### Period 4

Observed and randomly expected values for period 4 (days 66 to 85) tend to be the lowest for the season in most estimates, except in the case of *I*_mates(adj)_ (Figure 7C) and *I*_mates(phen)_ (Figure 7D), which, as previously described for period 2, show the opposite trend when compared to other estimates. However, when the observed values of period 4 are divided by their random expectation, period 4 has the highest values in most cases except for *V*_mates_, *I*_mates_, *I*_mates(adj)_ and *I*_mates(phen)_ (Figures 6D, 7F–H). Near the end of the season the abundance of individuals per plant had dropped dramatically (Figure 3), and period 4 was the only one in which no copulas were recorded in some days (Figure 2). Smaller sample sizes thus result in much larger 95% bootstrap confidence intervals and no significant differences from random expectations are observed (except for *I*_harem_, Figure 7E), nor from O/RE ratios of the first three periods.

#### The total opportunity for sexual selection based on phenology of female receptivity

The contribution of the three components of the observed value of *I*_mates(phen)_ is different for each period and for the whole season. According to these values, the contribution of *I_mates(t)_ and *I_males(k)_ to *I*_mates(phen)_ is negligible when compared to the contribution of *I*_sex ratio_ (Table 2 and Figure 7D).

**Table 2:** Estimates of the opportunity for sexual selection based on female reproductive phenology in *L. undecimlineata*.

	*I*_sexratio_ +	*I_mates(t)_ −	*I_mates(k)_ =	*I*_mates(phen)_
Period 1	0.629	2.8e–05	2.6e–05	0.629
Period 2	0.062	1.7e–04	1.6e–04	0.062
Period 3	0.370	2.3e–05	2.2e–05	0.370
Period 4	0.405	1.3e–05	0.5e–06	0.405
Season	0.685	1.1e–05	9.e–06	0.685

The total opportunity for sexual selection based on phenology of female receptivity (*I*_mates(phen)_) is obtained as the sum of the opportunity for selection caused by temporal variation in sex ratio (*I*_sexratio_) and the opportunity for selection caused by the variance in mating success among males averaged over time and weighed by sex ratio (*I_mates(t)_), and then substracting the opportunity for selection caused by the average temporal variance in mating success per male (*I_males(k)_).

### The Opportunity for Sexual Selection for the Whole Mating Season

The value on the extreme right in all graphs of Figures 5, 6, 7 show estimates for the whole season. In four cases, *m*, m*, *V*_harem_, *V*_mates_ (Figures 5A,C and 6A,B), the observed value is significantly higher than any particular period. In remaining cases (except for *I_mates(phen)_*, Figure 7D) the 95% bootstrap confidence intervals for the whole season overlap with confidence intervals of only 1 or 2 periods. When divided by their randomly expected values, all estimates overlap with at least two periods and in all cases, with the exception of *t* (Figure 5F), they are significantly higher than random expectations.

### A Quantitative Description of the Mating System

**Figure 8:**
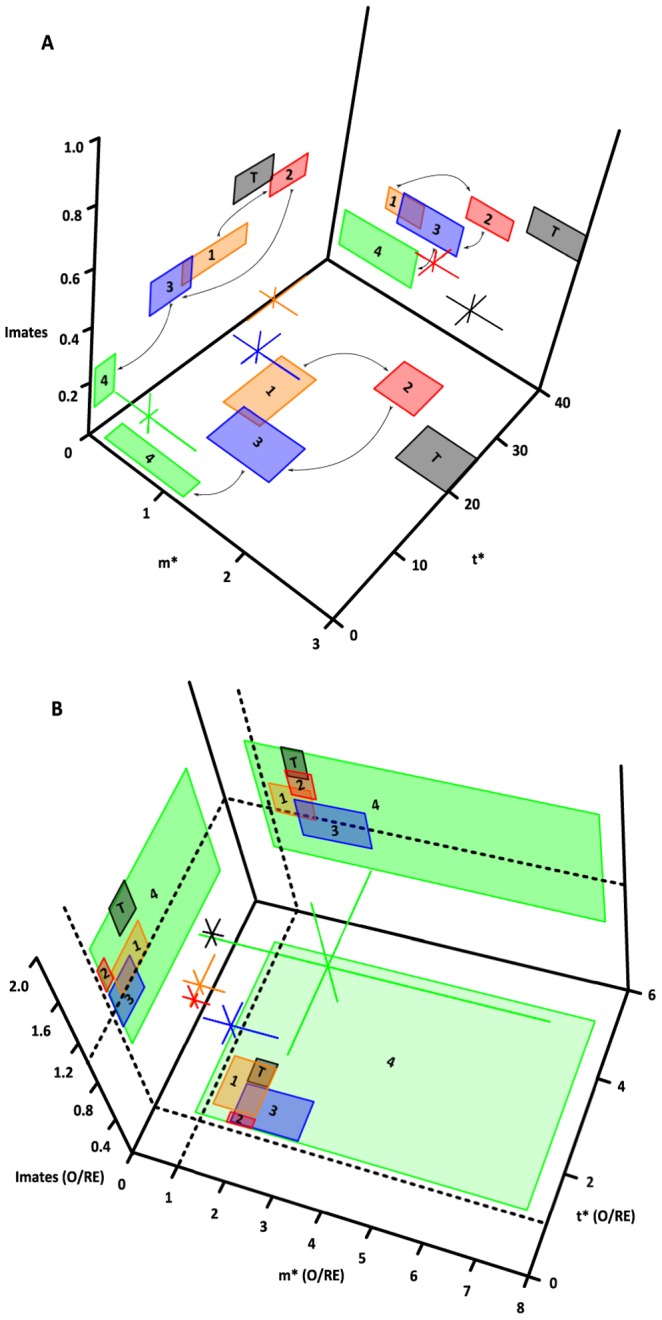
Quantitative characterization of the mating system of *L. undecimlineata*. The relationship between female mean crowding around males (m*), in time (t*), and *I*_mates_ for each period (1 in orange, 2 in red, 3 in blue and 4 in green) and the whole season (T in black). Orthogonal lines and projected squares represent bootstrap 95% confidence intervals for each value. **A**) Observed values following Shuster & Wade ([4], pages 92, 93). Arrows represent the temporal trajectory of the mating system throughout the season. High values of m* and t* in period 2 correspond with high values of *I*_mates_. **B**) The ratio between observed and randomly expected values (O/RE). The dashed horizontal lines at y = 1 in all planes represents no difference between observed and randomly expected values (O/RE  = 1). Only the difference of *I*_mates_ between periods 2 and 3 is significant, while only the values for period 2 and the whole season are significantly higher than random expectations.

Following Shuster and Wade ’s ([4] pages 92 and 93) attempt to represent quantitatively Emlen and Oring’s [3] verbal model relating the distribution of female receptivity in time and space with the strength of sexual selection, Figure 8 shows a three-dimensional representation of the relationship between mean spatial (m*) and temporal (t*) female crowding around males and the opportunity for sexual selection, *I*_mates_ (an interactive version of these graphs that may be rotated by the user using the mouse is available by running the R script provided in File S8 of Supporting Information). Consistent with these authors, *I*_mates_ is higher when observed values of m* and t* are high (period 2 in red and the whole season in black) than when both these values are low (period 4 in green). Periods 1 (in orange) and 3 (in blue) have intermediate values of m* and t* thus intermediate values of *I*_mates_. The significant differences marked by the 95% bootstrap confidence intervals (projected as boxes in the three planes) reveal a dynamic pattern in which the mating system starts the reproductive season at an intermediate value of *I*_mates_, increases in period 2, returns to an intermediate value in period 3 and drops to the lowest value at the end of the reproductive season (Figure 8A).

Nevertheless, once the observed values are divided by their expected value under random mating (Figure 8B), all four periods and estimates for the whole season overlap much more. The 95% bootstrap confidence intervals of period 4 engulf all the remaining periods, and even among those periods, only the difference of *I*_mates_ between periods 2 and 3 is significant, while only the values for period 2 and the whole season are significantly higher than random expectations (black doted lines on the three planes).

### Temporal Covariance in Male Mating Success and Sex Ratio

**Figure 9:**
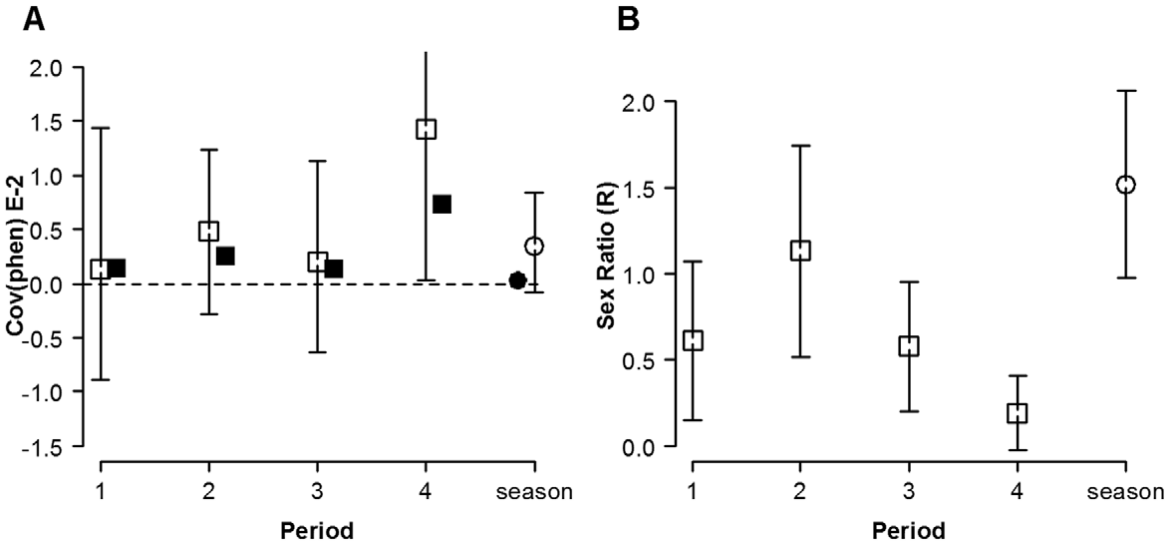
Covariance across temporal intervals in male mating success and sex ratio in *L. undecimlineata.* Observed values represented by open markers, randomly expected values represented by solid markers, 95% bootstrap confidence intervals represented by lines and random expectations represented by dashed horizontal line at y = 0. **A**) Covariance across temporal intervals in male mating success which includes the temporal and spatial variation of sex ratio (*Cov*_(phen)_ ×10^−2^). Only the observed value for period 4 differed from random expectations although no differences were observed among periods or to the whole season. **B**) Sex ratio (R), the number of receptive females to total males; there were no significant differences among periods while the value for the whole season was significantly different from periods 3 and 4.

The covariance among time intervals in male mating success (*Cov*_(phen)_) had the highest observed value in period 4 (open squares in Figure 9A), which is also significantly higher than random expectations (*i.e*., covariance  = 0). Nevertheless, the 95% bootstrap confidence intervals did not show any significant difference among periods nor with the whole season. Randomly expected values from the null model were consistently higher than 0 (solid squares in Figure 9A). In the case of *R* (receptive females/total number of males), period 2 showed the highest value, although it was only significantly higher than period 4 (Figure 9B).

### Male-male Precopulatory Competition Throughout the Season

**Figure 10:**
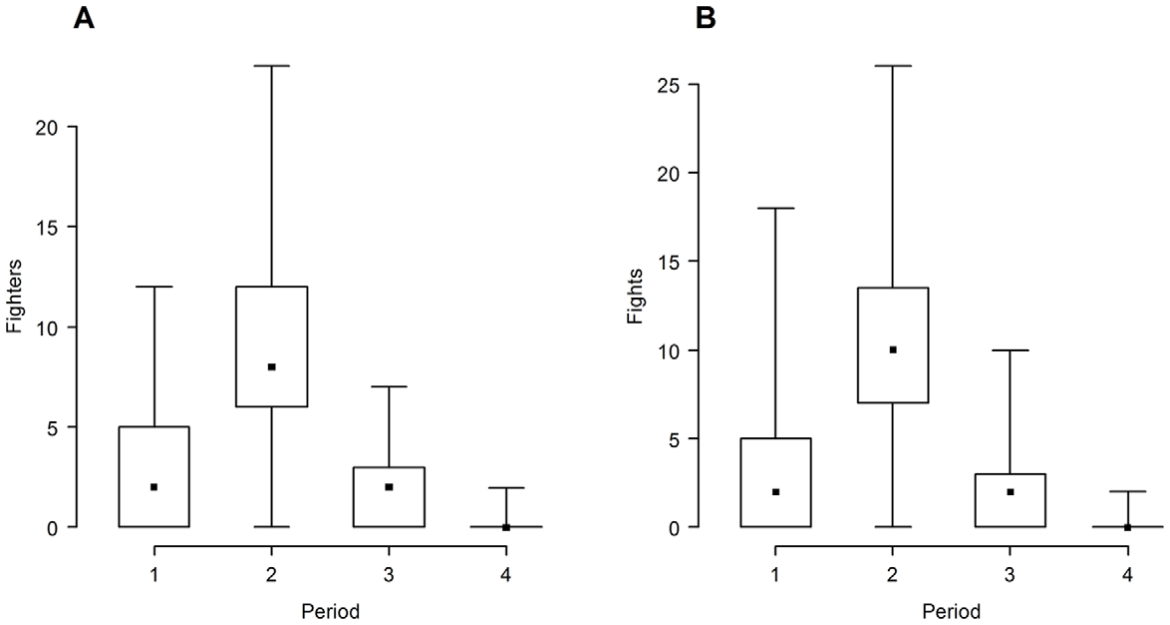
Male fighting in *L. undecimlieneata.* Fighter (**A**) and fight (**B**) frequency throughout the reproductive season (n = 20 days in all periods). Median, quartiles and extreme values are represented. Both the number of fighters and fight frequency peaked in period 2 (Tables 3,4).

With respect to the number of fighting males, only *Period* remained as a significant factor in the minimum model explaining 57% of the null deviance (Table 3). For the number of fights, not only was *Period* a significant factor, but also the number of occupied *Plants* and number of fighting males (*Fighters*), accounting for 83% of the null deviance (Table 4). However, *Period* by itself explained 56% of the model deviance. Both the number of fighting males and the number of fights peaked in period 2 (Figure 10A,B). Neither female nor male abundance per plant were significant factors in the number of fighting males or fights throughout the reproductive season.

**Table 3:** Minimal GLM model for number of fighting males in a population of *L. undecimlineata.*

Factor	d. f	Deviance	Explained deviance	P
Period	3	264.84	57%	<0.001
*Null*	*79*	*468.07*		
*Residual*	*76*	*203.23*	*43%*	

See Figure 10A.

**Table 4:** Minimal GLM model for number of fights in a population of *L. undecimlineata.*

Factor	d. f	Deviance	Explained deviance	P
Period	3	318.41	56%	<0.001
Plants	1	19.18	3%	<0.001
Fighters	1	135.64	24%	<0.001
*Null*	*79*	*572.81*		
*Residual*	*74*	*99.57*	*17%*	

See Figure 10B.

## Discussion

A marked temporal variation could be observed even in basic descriptive parameters such as copulas per male or overall abundance (Figures 2 and 3). These parameters started at intermediate values in period 1, peaked in period 2, returned to intermediate values in period 3, and collapsed in period 4. This pattern was mirrored by fluctuations in the observed values of all four estimates of the opportunity for sexual selection (Figure 7), as well as by many of their associated parameters (Figures 5 and 6). Furthermore, the *standardized mating differential* (*m*’, the covariance between a male trait and his mating success) was significantly different from 0 (the randomly expected value for such estimate) for male mobility in the middle of the mating season, but not for male size, showing how estimates of the opportunity for sexual selection may or may not predict estimates of the strength of sexual selection on phenotypic traits, and thus provide complementary information on the selective forces derived from ecological and social factors [5]. All four estimates of *I* for period 2, and two for period 3, are significantly higher than random expectations (Figure 7E–H), and more successful males either on male-male or male-female interactions are less likely to change plant than those less successful in the same periods (Figure 4B). This would be expected in a resource defense mating system but contradicts previous findings that male mobility is positively correlated to mating success in scramble mating competition mating systems [34]. However, as discussed below, there is no territorial defense in this population; as far as we know, such mating strategy is extremely rare or absent in chrysomelids. It is also worth noticing that the three estimates of *I_mates_* (Figure 7F–H) for period 1 are also significantly larger than their randomly expected values, but such opportunity for sexual selection does not result in significant selection for mobility. However, none of the estimates of *I_mates_* for period 4 are different from random expectations, thus it is to be expected that mating differentials for size and mobility are not significantly different from 0. The relatively low values of the three estimates of *I*_mates_ (Figure 7B–D and 7F–H) are within range of previous reports for other natural systems (reviewed in [5]). Little opportunity for sexual selection is to be expected in low monopolization mating systems such as scramble mating polygyny, compared to mating systems of high monopolization such as resource or female defense polygynies [4].

The fact that estimates of the opportunity for sexual selection depend not only on variance in male mating success but on male density and mean mating success has recently been highlighted [23], as well as the use of null models to account for the independent effect of each of these factors [2], [6]. It is worth describing why such estimates are sometimes said to be “biased”. Since not only male mating variance, but male density and average male mating success (or operational sex ratio) influence estimates of the opportunity for sexual selection, the effect of the variance in mating success cannot be separated from the effect of mean mating success and male density unless the expected value under random mating for a given number of males and copulas is known [6]. Our null models shuffle each period matrix (or the matrix including the whole season), but the total number of males and copulas (and thus average male mating success) do not change. Therefore, we obtain the variance within successful males (V_harem_) and between successful and unsuccessful males (V_mates_) expected from a given average male mating success and number of males in each of the four periods (or throughout the whole mating season), as well as the opportunity for sexual selection expected under random mating. The ratio between the observed and randomly expected values (O/RE) measures the effect of female aggregation increasing or decreasing the randomly expected opportunity for sexual selection. For example, O/RE  = 2 means that the effect of female aggregation doubles the opportunity for sexual selection expected under random mating for a given average male mating success and male density. Our results revealed that randomly expected values per period also fluctuate, as would be expected due to variation in male density and average mating success, but only in some cases such fluctuation closely follows the observed values. Thus, the effect of aggregation of female receptivity on *I*_mates_ may be extremely variable along the reproductive season as shown by our O/RE ratios. Other estimates such as the index of resource (females) monopolization (*Q*), Morisita’s index (*Iδ*) and the standardized Morisita index (*Ip*) [1], [21], [22] attempt to control for such random expectation algebraically. However, their interpretation is not directly related to evolutionary theory [1], [23], and we suggest that comparing each parameter derived from evolutionary theory with its own randomly expected value is a more solid alternative. In fact, there is a similar rationale behind selection (or mating) differentials in which the randomly expected value is 0.

As discussed above, it is worth noticing that substantial fluctuation among all parameters under random mating is to be expected solely as result of fluctuation in male density and mean male mating success (solid squares in Figures 6 and 7). This shows how much opportunity for sexual selection may be expected in each period purely by male abundance and average mating success or by operational sex ratio, regardless of any variance in male mating success. This may explain why the randomly expected fluctuation in the opportunity for sexual selection derived from female aggregation around successful males (*I*_harem_) or from aggregation around all males (*I*_mates_) was similar (Figure 7A,B). The causal relationship between density, sexual selection and mating strategies has been somewhat ignored and the need for empirical work exploring these relationships has been stressed [47]. In particular, the role of male density on population dynamics and mating systems has been specially overlooked [48].

Emlen and Oring [3] also suggested that female synchrony (receptive females aggregated in time) and the number of sexually active males relative to the number of sexually active females in a population (Emlen and Oring’s [3] OSR and Shuster and Wade’s [4] *R*_o_) are directly correlated with the intensity of sexual selection. Here, female synchrony is estimated by t*, and average number of mates per male by sex ratio (*R*) instead of OSR (Ro) since Shuster and Wade’s [4] model focuses on aggregation of female receptivity around males. Period 2 had the highest values in both t* (Figure 5D) and *R* (Figure 9B), and thus expected and observed values of *I*_mates_ also peaked in period 2 (Figure 7B,F). However, the last two estimates of the opportunity for sexual selection, *I*_mates(adj)_ and *I*_mates(phen)_, (Figure 7C, D) had the lowest randomly expected values in the same period (Figure 7C,D). Considering all this, our study shows how estimates of *I*_mates_ that do not consider the influence of sex ratio or female aggregation in time may present a completely different picture than estimates which do consider those factors. Then, it seems that the effect of sex ratio and female aggregation in time overrides the effect of female aggregation around males and variance in mating success in this population.

The actual (observed) values of all these estimates, when divided by their randomly expected values, provided additional insights. Period 2 had values significantly larger than expected under random mating in all estimates of *I* (Figure 7E–H), although they overlapped with O/RE ratios for periods 1 and 3. Variances in male mating success among successful males (*V*_harem_, Figure 6C), and among all males (*V*_mates_, Figure 6D) were significantly higher than expected under random mating for period 2, barely higher for period 1, and not different or even significantly lower than random expectations in period 3. This seemed to result in opportunities for sexual selection (*I*_harem_ and *I*_mates_, Figure 7E,F) not significantly different from random expectations in period 3, barely higher for period 1, and significantly higher for period 2. The opportunity for sexual selection for both estimates that factor in sex ratio resulted in significantly higher randomly expected values for all three periods, thus highlighting the contribution of female aggregation. Relatively fewer mating events in period 4 due to fewer individuals in the population and a decrease in reproductive behavior resulted in much larger confidence intervals thus limiting opportunities to compare with other periods and highlighting how nonsignificant results may be due to naturally smaller sample size due to lower densities at the end or beginning of a mating season. The need to consider sample size when interpreting estimates of sexual selection has been previously stressed [49].

The strong effect of sex ratio may also be observed when the opportunity for sexual selection derived from phenology of female receptivity (*I*_mates(phen)_) is divided in its three components (Table 2). The estimate derived from sex ratio (*I*_sex ratio_) is several orders of magnitude larger than the other two components; the weighted opportunity for sexual selection at any particular time interval (*I_mates(t)_), and the weighted opportunity for sexual selection within males (*I_males(k)_). *I*_mates(phen)_ focuses on how female receptivity in time influences sex ratio when daily mating success of each male accumulates in time. Although *I*_mates(phen)_ values for periods 1, 2 and 3 were significantly higher than random expectations, period 2 was significantly lower than period 1 (Figure 7H). This is most likely due to the fact that copulas were more evenly distributed in time during period 1 than expected by chance (high synchrony) as can be expected from high values of average number of mated females per time interval (*t*) and temporal female crowding (t*) (Figure 5D–H). Even though there were relatively more females in copula in period 2, probably due to the peak in oocite maturation [37], the value of *I*_mates(phen)_ was significantly higher than the randomly expected value suggesting that a few males accumulated more mating events than would be expected under random mating.

The covariance among time intervals in individual male mating success, *Cov*_(phen)_, is the degree to which only a few males accumulate mating events over time intervals ([4]; page 90). Surprisingly, its value (open squares in Figure 9A) was not significantly different from 0 in periods 1, 2 and 3, although their values for *I*_mates(phen)_ were significantly larger than random expectation. Furthermore, *Cov*_(phen)_ for period 4 was significantly higher than 0, even though only in this period *I*_mates(phen)_ was not significantly higher than random expectations. It is worth noting that randomly expected values for *Cov*_(phen)_ from the null model (solid markers in Figure 9A) were larger than 0 in all periods, thus suggesting a slight bias in this estimate as the randomly expected value for any covariance is 0.

The relative values of spatial and temporal mean crowding are diagnostic of a mating system [4] since the distribution of number of mates per male over the mating season is one of the main descriptive traits of mating systems [3]. When spatial mean crowding is high we expect a resource defense based mating system. In our case, however, temporal mean crowding is an order of magnitude larger than spatial mean crowding around males, thus suggesting that female aggregation in time is a more important element in this mating system, as would be the case in a *scramble competition polygyny* [29]. Even though host plants are discrete resources and both males and females usually stay on a single plant for several days (Baena and Macías-Ordóñez, unpublished), there is no territorial defense of plants, sections of plants, or precopulatory mate guarding, although males often fight or stay with and defend ovipositing females immediately after mating (Baena and Macías-Ordóñez, unpublished). Host plants do not seem to be economically defendable due to their structure and large size as females move freely among leaves feeding, mating and ovipositing (Baena and Macías-Ordóñez, unpublished).

Shuster and Wade [4] suggest that intermediate to high values of spatial and temporal mean crowding (m* and t*) promote male mate guarding, larger and more aggressive successful males, territorial defense and frequent fights. These predictions are aimed to contrast different populations or species, although a similar approach should hold for seasonal changes within the same population. In our study, high values of m* and t* in period 2 correspond with high values of *I*_mates_ (Figure 8A), and with more fights (Figure 10B). However, once these values are divided by their random expectations (Figure 8B), all four periods and estimates for the whole season show much more overlap. In other words, it seems that a large component of the dynamic fluctuations among periods is due to fluctuations merely in number of males, females and copulas (to which m* and t* are also clearly sensitive); and a much smaller fraction seems to be due to actual changes in non-random female aggregation (or monopolization by males) in time or space.

Although there were significantly more fights and fighting males in period 2, no fights were observed when males remained with egg laying females after mating and nothing resembling territorial defense was observed. Thus, it seems that more fighting activity in period 2 may have been simply the result of higher male density and not evidence of stronger intra-sexual selection. Once sex ratio and phenology of female receptivity are accounted for, the opportunity for sexual selection is not significantly higher in period 2, and is even lower than period 1 (*I*_mates(adj)_ and *I*_mates(phen)_, Figure 7C,D). Furthermore, the mating differential (*m* ´) based on male size, a likely predictor of fighting success, was not significantly higher than 0 in any period (Figure 4A). Thus, as expected under scramble competition [50] and as observed in other chrysomelids [34], our results do not suggest a male mating advantage related to size.

The sharp fluctuations in many parameters throughout the reproductive season of *L. undecimlineata* suggest that not only is this type mating system hard to define or pinpoint in the conceptual framework of Emlen and Oring [3], but its actual reproductive dynamics are highly plastic and context dependent. Unlike *leks*, in which females are not predictable nor are males able to monopolize them, or *resource (or female) based polygynies* in which resources (or females) are both predictable and may be monopolized, in the case of scramble competition polygyny males can predict resources females seek but cannot monopolize them [29].

Estimating parameters of the mating system in the absence of a temporal scale may provide a picture that does not represent any given period within the reproductive season. Choosing the appropriate time scale is crucial to understanding the dynamics of this and many other mating systems. Trying to define a mating system as a temporally homogeneous system hides the actual change in mating strategies that define each period, in which different ecological circumstances may produce different sex specific selection pressures. Furthermore, assessment of the opportunity for, or the strength of, sexual selection for long periods assumes that all males observed were present throughout the period, in which case males that left the population or died are considered unsuccessful despite their absence, and thus the variance in mating success would be overestimated (see [7] for discussion on when absent males should be included).

The use of null models and resampling methods to assess the actual statistical and biological significance of population parameters is uncommon, especially in parameters that define the mating strategies and reproductive phenology of populations. The use of estimates of the intensity of and opportunity for sexual selection is subject to much discussion in the literature [2], [5], [6], [23], [27], [45] and it has recently been suggested that, without comparing such estimates with the values expected by chance, it is hard to assess the relative contribution of sample size, mean and variance in mating success [2], [6]. The differences and similarities between estimates of *the opportunity for sexual selection*, or between these and mating differentials, highlight the importance of using them all when possible, in order to pinpoint the effects of different behavioral, ecological or demographic effects behind each estimate, see [2], [51].

Few studies, if any, have quantitatively defined mating system and compared the assessment of the opportunity for and strength of sexual selection at different temporal scales [52]. Temporal fluctuations observed in most parameters of the scramble competition polygyny of *L. undecimlineata* suggest that the opportunity for sexual selection and the frequency of male-male fights vary widely throughout the mating season, and these changes are followed by the strength of sexual selection either on a behavioral trait (mobility), or on traits correlated to it, but not on size. Scramble mating polygyny is probably the most common mating system among insects [29] and the name itself is suggestive of weak or absent sexual selection. Although it has been suggested that sexual selection (or mating) differentials are better predicted by the opportunity for sexual selection in other polygynous mating systems such as resource defense polygyny or female defense polygyny [7], our study suggests that they may covary even on mating systems with low or no monopolization if the right trait is identified. More importantly, however, is to recognize that estimating both the opportunity for and the strength of sexual selection, and ideally their temporal fluctuations, provides a much more complete picture.

## Supporting Information

File S1 **A worked example.** Hypothetical data of six time intervals and twenty males showing how to obtain all parameters related to three estimates of the opportunity for sexual selection (*I*_mates_) presented in Shuster, S. M., and M. J. Wade. 2003. Mating Systems and Strategies. Princeton (NJ): Princeton University Press, chapters 1–3. Some of the steps were more fully developed by personal communications with S. Shuster. Double click on matrix to activate spreadsheet.
(DOC)

File S2 **R script for null model for** ***I*** **_mates_ and related parameters.** A null model generator for *I*_mates_ and related parameters based on Shuster, S. M., and M. J. Wade. 2003. Mating Systems and Strategies. Princeton (NJ): Princeton University Press, chapters 1–3. Observed data should be in a csv file (named “File S3 - Matrix of mating success per time interval per male for the whole season.csv” in this case) containing only 0′s & 1′s (mating events), one male per row, one time interval (e.g. 1 day in this study) per column. No column or row names should be included. See script for instructions to test any other.csv matrix.
(R) Click here for additional data file.

File S3 **Matrix of mating success per time interval per male for the whole season.** This database in.csv format is required by “File S2. R script for null model for Imates and related parameters.r” (see description in legend for File S2). It should be placed in the same working directory of File S2.
(CSV)

File S4 **Matrix of mating success per time interval per male for period 1.** In order to see results for period 1 only instead of results for the whole season, this database in.csv format may replace “File S3 - Matrix of mating success per time interval per male for the whole season.csv” when running “File S2. R script for null model for Imates and related parameters.r” (see script for instructions). It should be placed in the same working directory of File S2.
(CSV)

File S5 **Matrix of mating success per time interval per male for period 2**. In order to see results for period 2 only instead of results for the whole season, this database in.csv format may replace “File S3 - Matrix of mating success per time interval per male for the whole season.csv” when running “File S2. R script for null model for Imates and related parameters.r” (see script for instructions). It should be placed in the same working directory of File S2.
(CSV)

File S6 **Matrix of mating success per time interval per male for period 3.** In order to see results for period 3 only instead of results for the whole season, this database in.csv format may replace “File S3 - Matrix of mating success per time interval per male for the whole season.csv” when running “File S2. R script for null model for Imates and related parameters.r” (see script for instructions). It should be placed in the same working directory of File S2.
(CSV)

File S7 **Matrix of mating success per time interval per male for period 4.** In order to see results for period 4 only instead of results for the whole season, this database in.csv format may replace “File S3 - Matrix of mating success per time interval per male for the whole season.csv” when running “File S2. R script for null model for Imates and related parameters.r” (see script for instructions). It should be placed in the same working directory of File S2.
(CSV)

File S8 **R script for interactive** **Figure 8** **.** Install the rgl library first. Then run this R script from beginning to end and two windows will appear (panels A and B). Amplify or maximize each window, click and hold on the graph and then move the mouse to rotate it. You will be able to see the projections on the three planes. See figure 8 for legend and the text for more information.
(R) Click here for additional data file.
