# Correction: Phenology of Scramble Polygyny in a Wild Population of Chrysolemid Beetles: The Opportunity for and the Strength of Sexual Selection

**DOI:** 10.1371/annotation/16cca963-5168-485d-8187-a435b12befde

**Published:** 2012-10-05

**Authors:** Martha Lucía Baena, Rogelio Macías-Ordóñez

There was a typographical error in the title and citation. The correct title is: Phenology of Scramble Polygyny in a Wild Population of Chrysomelid Beetles: The Opportunity for and the Strength of Sexual Selection. The correct citation is:

Baena ML, Macías-Ordóñez R (2012) Phenology of Scramble Polygyny in a Wild Population of Chrysomelid Beetles: The Opportunity for and the Strength of Sexual Selection. PLoS ONE 7(6): e38315. doi:10.1371/journal.pone.0038315

