# Supplementary material for: Phenology of Scramble Polygyny in a Wild Population of Chrysolemid Beetles: The Opportunity for and the Strength of Sexual Selection
Source: PLoS One. 2012 Jun 25;7(6):e38315. doi: 10.1371/journal.pone.0038315 (PMC3382595; doi:10.1371/journal.pone.0038315)
Supplement: File S1 — A worked example. Hypothetical data of six time intervals and twenty males showing how to obtain all parameters related to three estimates of the opportunity for sexual selection (I mates) presented in Shuster, S. M., and M. J. Wade. 2003. Mating Systems and Strategies. Princeton (NJ): Princeton University Press, chapters 1–3. Some of the steps were more fully developed by personal communications with S. Shuster. Double click on matrix to activate spreadsheet. (DOC) [file pone.0038315.s001.doc]

**File S1 - A worked example.** Supporting Information for Baena M. and Macías-Ordóñez R. 2012. Phenology of Scramble Polygyny in a Wild Population of Chrysolemid Beetles: The Opportunity for and the Strength of Sexual Selection. PLoS ONE.

A WORKED EXAMPLE

Hypothetical data of six time intervals and twenty males showing how to obtain all parameters related to three estimates of the opportunity for sexual selection (*I*_mates_) presented in Shuster, S. M., and M. J. Wade. 2003. Mating Systems and Strategies. Princeton (NJ): Princeton University Press, chapters 1-3. Some of the steps were more fully developed by personal communications with S. Shuster.

**Double click on matrix to activate spreadsheet.**

**∑m_i_** (Total number of copulas or receptive females in the *i*th patch of resource [or in the *i*th male]) =13

**∑(m_i_)^2^** = 51

**M** (Number of resource patches [or males] containing at least one female) =5

**N _Male total_** (Total number of males) = 20

**m** = ∑m_i_/M (Average density per patch) = 13/5 = 2.6

**V_m_** = (∑(m_i_)2/M) – m^2^ (Variance of density among patches) = (51/5) – (2.6)^2^ = 3.44

**m*** = (m + (V_m_/m)- 1) (mean spatial crowding of receptive females) = (2.6 + (3.44/2.6) – 1) = 2.92

**P** = m*/m (Patchiness) = 2.92/2.6 = 1.12

**H_mates(m)_** = ∑m_i_/M (= **m**. Average harem size of successful males) = 13/5 = 2.6

***V*_mates(m)_** (Variance in mate numbers among successful and unsuccessful males) = ((∑(m_i_)^2^ - (∑m_i_^2^/N_Male_ _total_))/N_Male_ _total_) = ((51-(13)2/20))/20) = 2.12

***I*_mates_** = *V*_mates(m)_ /H^2^ (Opportunity for sexual selection) = 2.12/(2.6)^2^ = 0.314

***V*_harem (m)_** = ((∑(m_i_)^2^ - (∑m_i_^2^/M))/ M ) (Variance in mating success among successful males) = ((51- (13)2/5))/5) = 3.44

***I* _harem(m)_** = *V*_harem(m)_ /H^2^ (Opportunity for sexual selection on successful males) = 3.44/(2.6)^2^ = 0.508

**∑t_i_** (Number of copulas or receptive females in the *i*th interval) =13

**∑(t_i_)^2^** = 31

**T** (Number of time intervals containing at least one receptive female) = 6

**t =** ∑t_i_ / T (Average number of copulas or receptive females over all *i* intervals) = 13/6 = 2.17

**V_t_** = (∑(t_i_)2/T) – t^2^ (Temporal variance in the number of copulas or receptive females) = (31 / 6) – 2.17 = 0.472

**t*** = (t + (V_t_/t)- 1) (mean temporal crowding of copulas or receptive female) = (2.17 + (0.472/2.17) – 1) = 1.38

**S** = t*/t (temporal patchiness of female breeding phenology ) = 1.38/2.17 = 0.64

**P_o_** = 1- (M/T) (Fraction of unsuccessful males) = 1 – (5/20) = 0.75

**1-P_o_** (Fraction of successful males)= 1-0.75 = 0.25

**N males (1-Po)** = N _male total_ * (1-P_o_) (Number of successful males) = 20 * 0.25 = 5

**H_mates(t)_** = ∑t_i_/ N _male total_ * (1-P_o_) (Average harem size of successful males) = 13/5 = 2.6

***V*_mates (t)_** = ((∑(m_i_)^2^ - (∑m_i_^2^/ N_Male_ _total_))/( N_Male_ _total_) (Variance in mate number of successful and unsuccesfull males) = ((51 - (13)2/20))/20) = 2.12

***I*_mates(t)_** = *V*_mates (t)_ /H_mates(t)_ (Opportunity for sexual selection) = 2.12/(2.6)^2^ = 0.314

***V*_harem(t)_** = ((∑(m_i_)^2^ - (∑m_i_^2^/M))/(M) (Variance in mating success among successful males) = ((51 - (13)2/5))/5) = 3.44

***I* _harem(t)_** = *V*_harem (t)_ /H_mates(t)_ (Opportunity for sexual selection on successful males) = 3.44/(2.6)^2^ = 0.508

**t_i_**  (R_t_) = **∑** R(t) = (The individual ratios of copulas or receptive females to males obtain per day) = 0.65

**R_O_** = (Operational sex ratio) = N_males (total)_ / **∑m_i_** = 20/13 = 1.54

**(ΣV_mates[t])_ /T) =** (Average variance in male mate numbers across all intervals = the within interval variance in mating success) = 0.5725/6 = 0.0954

**V(R[t]) =** (Variance of the average male mate numbers among intervals across the breeding season= the among interval variance in mate numbers) **= VARP N._j_ /K** = 0.0118

**V(bar)_t_ (k)**  = (Average variance of mating success for successful males. It measures the ups and downs of mating success experienced by the average male) = 0.0375

**Vmates/(T)** : (Variance among rows in the average number of mates per male): VARP ∑mi /T = 7.94 (Table 1, this appendix).

**Cov _(mates)_** (Covariance among time intervals in male mating success) = (ΣVmates[t])/T - V(R[t]) = 0.0954 – 0.0118 = 0.0836

***I*_sexratio_** (Opportunity for sexual selection caused by temporal variation in the sex ratio) = V(R[t]) * (T/t_i_ (R_t_)) ^2^ = 0.0118 * (6/0.65)^2^ = 0.10059

****I*_mates(t)_** (The weighted opportunity for sexual selection caused by temporal variation in the availability of females) = (ΣVmates[t])/T * (t_i_ (R_t_)/T) ^2^ = 0.095 * (0.65/6)^2^ = 0.0011

****I*_males(k)_** (The weighted opportunity for sexual selection caused by spatial variation in the availability of females) = V(R[t]) * (t_i_ (R_t_)/T)^2^ = 0.0118 * (0.65/6)^2^ = 1.38 e-5

***I*_mates(phen)_** (Opportunity for sexual selection caused by receptive phenology of females)

= * *I*_sexratio_ + **I* _mates(t)_ - **I* _mates(k)_ = 0.10059 + 0.0011 – 1.38 e-5 = 0.1017
